# QSAR-Based Ecotoxicological Assessment of Novel Imidazole Derivatives for Sustainable Crop Protection

**DOI:** 10.3390/molecules31183166

**Published:** 2026-09-09

**Authors:** Gabriella Kanižai Šarić, Marija Paurević, Andrea Dandić, Martina Šrajer Gajdošik, Vesna Rastija

**Affiliations:** 1 Faculty of Agrobiotechnical Sciences Osijek, Josip Juraj Strossmayer University of Osijek, Vladimira Preloga 1, 31000 Osijek, Croatia; gabriella.kanizai@fazos.hr; 2 Department of Chemistry, Josip Juraj Strossmayer University of Osijek, Cara Hadrijana 8A, 31000 Osijek, Croatia; mstivo@kemija.unios.hr (M.P.); andreajuric@kemija.unios.hr (A.D.); msgajdosik@kemija.unios.hr (M.Š.G.)

**Keywords:** pesticide, imidazole, cheminformatic, QSAR, ecotoxicology, pesticide-likeness, molecular docking

## Abstract

Imidazoles have been proven to be very effective pesticides, especially against phytopathogenic fungi and insects. Due to their negative effects on the environment, only a few imidazoles have been approved for use by the European Commission (EC). There is an urgent need to develop new imidazole derivatives with high efficiency and a wide spectrum of action against numerous pests that are, at the same time, safe for the environment and beneficial for organisms and humans. In order to reduce expensive and time-consuming experiments, an *in silico* approach based on quantitative structure–activity relationship (QSAR) models is valuable for predicting the toxicity of new or untested chemicals. In this study, we used the Vega and ChemFREE web platforms to evaluate the pesticide similarity, environmental risk properties, and ecotoxicological effects of imidazole derivatives designed for potential synthesis. Adamantane-, alkyl-, and triazole-amide, ester, carbamate, and ketone derivatives were filtered for the evaluated properties, and four alkyl-amides were highlighted as potentially effective and environmentally safe antifungal, herbicidal, and insecticidal agents. Molecular docking studies indicated the possible mechanism of action of the antifungal, herbicidal, insecticidal, and antibacterial activities of the observed compounds and revealed structural features important for binding to specific receptors.

## 1. Introduction

The present use of synthetic compounds in plant protection has limited the occurrence and development of plant diseases and pests. Resistance to pesticides and their environmental and health hazards [1] indicate an urgent need for new active ingredients for plant protection products. These compounds must be highly specific and environmentally and toxicologically acceptable. The ecological effects of pesticides are influenced by their physicochemical properties, application rate, exposure duration, and environmental conditions, particularly soil physicochemical properties [2]. Persistence refers to the tendency of a pesticide to remain in an environmental compartment, such as soil, water, air, or sediment, before undergoing chemical or biological transformation [3]. Under suitable environmental conditions, soil microorganisms can utilize pesticides as nutrient sources, facilitating their degradation through diverse enzymatic pathways. These transformation processes are primarily mediated by microbial enzymes that convert pesticide molecules into intermediate metabolites, which may subsequently undergo further degradation [4]. Both persistence and biodegradation are strongly influenced by a compound’s chemical structure, which makes these endpoints particularly amenable to prediction through structure-based computational approaches [5].

Modern crop protection strategies increasingly demand the design of compounds that pair high biological activity with significantly reduced toxicity toward non-target organisms, thereby driving the transition toward sustainable agriculture. Regulation (EC) No. 1107/2009 of the European Parliament and of the Council concerning the placing of plant protection products on the market introduced stricter criteria for including active substances in the list of approved active substances for use in plant protection products. An active substance should not have any harmful effect on human or animal health or cause any unacceptable effects on the environment. In addition, the regulation promotes the use of non-animal testing methods and other risk assessment strategies regarding the protection of animals [6]. The discovery of pesticides in the laboratory and their subsequent commercialization is a cost-intensive and time-consuming process that delays the pesticide development process. Computer-aided molecular design (CAMD) has been generally accepted and extensively applied in the ecotoxicological modeling and design of agrochemicals owing to its high efficiency in the design of new compounds, saving both time and economic costs in large-scale experimental synthesis and biological tests. According to the REACH (Registration, Evaluation, and Authorization of Chemicals) guidelines of the European Chemicals Agency (ECHA) from 2011, animal tests can be avoided if the hazardous properties of a substance can be predicted using computer models as part of a quantitative structure–activity relationship (QSAR) approach (ECHA-11-R-004.2-EN) [7].

**Figure 1:**
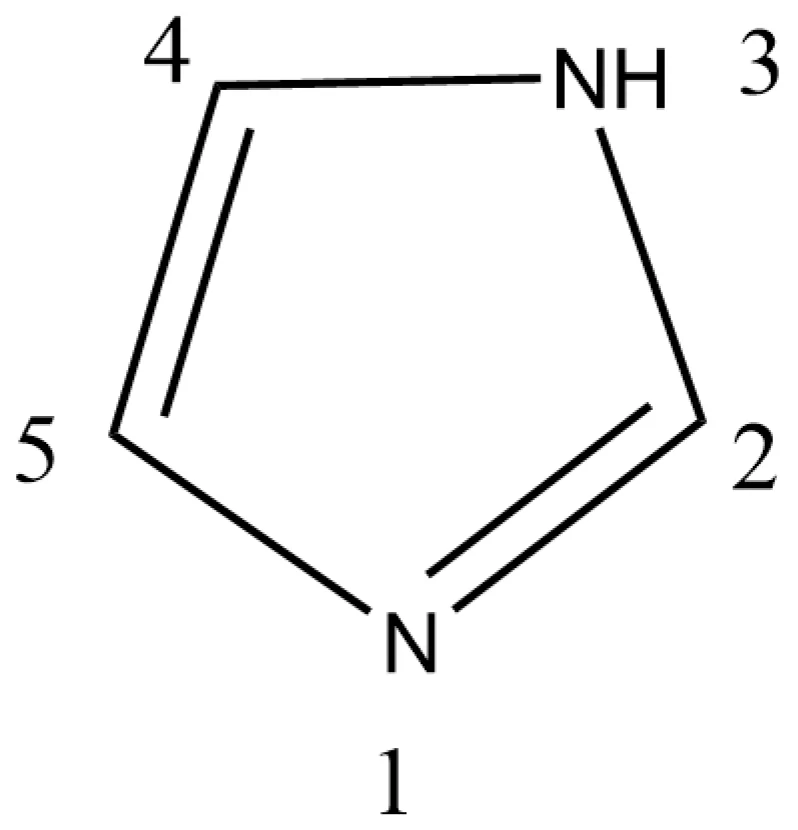
Imidazole core.

Imidazoles are aromatic molecules that consist of a five-membered heterocyclic aromatic ring with two nitrogen atoms clustered at positions 1 and 3 (Figure 1).

Imidazoles possess a broad range of pharmacological properties, such as antibacterial, antifungal, antiviral, antiamoebic, anthelmintic, anti-inflammatory, antitumor, antidiabetic, antiallergic, antipyretic, antioxidant, and ulcerogenic activities [8,9,10]. The imidazole ring shows excellent solubility in water and other polar solvents, which helps to overcome the issues with poorly soluble drug entities [9].

Among these diverse biological activities, the antifungal potential of imidazole derivatives has been particularly well exploited in agricultural applications, leading to the development of several imidazole-based fungicides for plant protection. However, only a limited number of fungicides remain approved for use within the European Union following periodic regulatory re-evaluation [11]. Among them, imazalil is one of the best-established representatives currently approved for specific plant protection uses, whereas the approval of prochloraz has not been renewed due to concerns regarding human health and environmental safety [12].

Imidazole-based compounds are still widely studied for their antifungal, antibacterial, antiviral, insecticidal, and nematicidal activities in the soil environment [13]. The ionic liquids based on the imidazolium cation have shown strong antifungal effects against *Fusarium* [14] and against *Alternaria* species [15]. Because imidazole fungicides inhibit fungal sterol biosynthesis, they can alter fungal community composition and indirectly influence bacterial communities by disrupting fungal–bacterial interactions and nutrient turnover [16].

Only a few toxicological studies have assessed the potential environmental effects of imidazole and its impact on organisms. For the pesticide imazalil, it was confirmed that it did not cause lethality to exposed organisms, but chronic exposure to this pesticide may lead to sub-lethal effects on the earthworm *Eisenia andrei*, related to reproduction, cytotoxicity, and oxidative stress [17]. It was also shown that higher concentrations of imidazole ionic liquids decreased the germination rate and reduced root and stem length while increasing the root and stem inhibition rate in two terrestrial plants [18].

There is an urgent need to develop new imidazole derivatives with high efficiency and a broad activity spectrum against a large number of pests, which are, at the same time, safe for humans, the environment, and beneficial organisms. Organic matter mineralization and nutrient cycling are among the ecological functions sustained by soil microbial communities [19]. Additionally, microorganisms contribute to plant resilience by enhancing resistance to biotic and abiotic stresses, activating plant defense responses, promoting soil aggregation, and facilitating xenobiotic degradation. Any disruption of microbial diversity or activity can affect soil health, weaken ecosystem resilience, and ultimately threaten long-term crop productivity [20,21]. Preserving these microbial functions therefore requires plant protection products that are both effective and minimally disruptive to non-target soil organisms. This is also reflected in current European agricultural policies, such as the Green Deal and the Farm to Fork Strategy, which recommend reducing reliance on conventional chemical pesticides in favor of safer alternatives [22].

To reduce expensive and time-consuming experiments, the quantitative structure–activity relationship (QSAR) method is valuable for predicting the toxicity of new or untested chemicals.

The objectives of this study are to estimate the pesticide-like properties of 32 derivatives of imidazole, as well as their ecological and environmental risks prior to potential synthesis. The designed compounds are alkyl-, adamantane-, and triazole-amide, ester, carbamate, and ketone derivatives of imidazole. In order to prevent the development of pesticide resistance in the target pest(s), it is important to understand the pesticides’ mechanism of action. A molecular docking study was thus performed on the enzymes whose action is related to a particular biological activity (antifungal, herbicidal, insecticidal, and antibacterial) in order to identify the possible mechanism of action. That knowledge will be used for the further synthesis and development of imidazoles as ecotoxicologically safe plant protection agents.

## 2. Results

Table 1 shows the predicted pesticide-likeness and environmental risk properties of the imidazole derivatives designed as potential plant protection agents.

Predicted ecotoxicological properties for the derivatives of imidazole are presented in Table 2.

The highest probability of becoming antifungal agents was observed in compounds with the highest value of RDL, such as three imidazole derivatives of adamantane (**6**, **7**, and **16**), and five alkyl derivatives (**18**, **27**, **28**, **29**, and **30**). The best herbicidal likeness properties were predicted for the derivative of adamantane **8** and for alkyl derivatives (**12**, **13**, **14**, **15**, **16**, **18**, **27**, **28**, **29**, **30**, **31**, and **32**). For a possible insecticidal effect, the highest probability was calculated for the derivative of adamantane **9** and for alkyl derivatives (**13**, **14**, **15**, **27**, and **31**). Apart from compounds **27**, **29**, and **31**, none of the mentioned compounds are biodegradable. Derivatives of adamantane are generally poorly biodegradable, and that is the key reason why they were initially eliminated. Due to being tricyclic hydrocarbons with a rigid, diamond-like structure, adamantanes are characterized by high stability and low susceptibility to microbial degradation [23].

The highest acute and short-term toxicity on aquatic animals was predicted for acetamide (**1** and **4**), carboxamide as iodide salts (**6** and **7**), and acetate (**9**, **10**, and **17**) adamantane derivatives of imidazole. For algae, the highest toxicity was predicted for derivatives with a chlorophenyl moiety in their structure (**21**–**24**).

The studied derivatives of imidazole are relatively safe for bees since they have low-to-moderate calculated toxicity toward this beneficial terrestrial arthropod. The triazole moiety, on the other hand, enhances the probability for toxicity against earthworms, especially for the imidazole derivative of 1,2,3-triazole (**19**).

Sludge toxicity implies the effects of a substance on microorganisms from activated sludge (largely bacteria) and is assessed by measuring their respiration rate. Since only substances with an experimental value predicted EC50 < 50 mg L^−1^ are considered “toxic”, the designed compounds could display toxicity on microorganisms in sludge. The highest toxicity was predicted for the ethyl ethyl carbonate (**13**) and methyl carbonate (**18**). The bioconcentration factor is closely related to the lipophilicity of compounds. Therefore, a high value of BCF was observed in the most lipophilic compound **10**, although imidazole salts (**5** and **6**), because of their excellent solubility in water (high hydrophilicity), could be found in high concentrations in aquatic organisms. According to the applicability domain of the QSAR models, most of the estimated values in Table 2 have low reliability of prediction and must therefore be considered with caution.

**Scheme 1:**
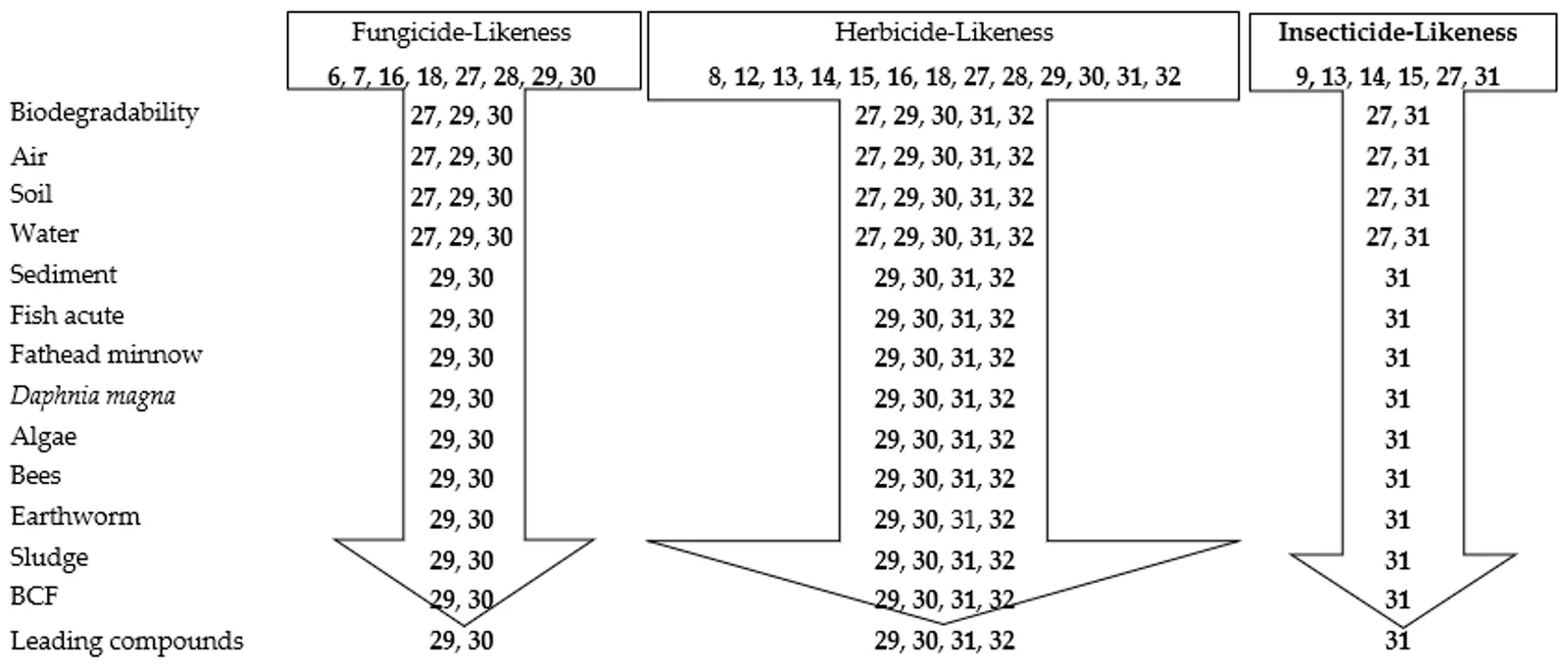
Schematic representation of the screening of potentially active substances of plant protection products through filters of environmental risk and ecotoxicological properties.

In order to summarize the obtained results and highlight the leading candidates, we filtered the compounds with the highest pesticide-likeness through calculated environmental risk and ecotoxicological properties. The algorithm of the procedure is presented in Scheme 1.

**Figure 2:**
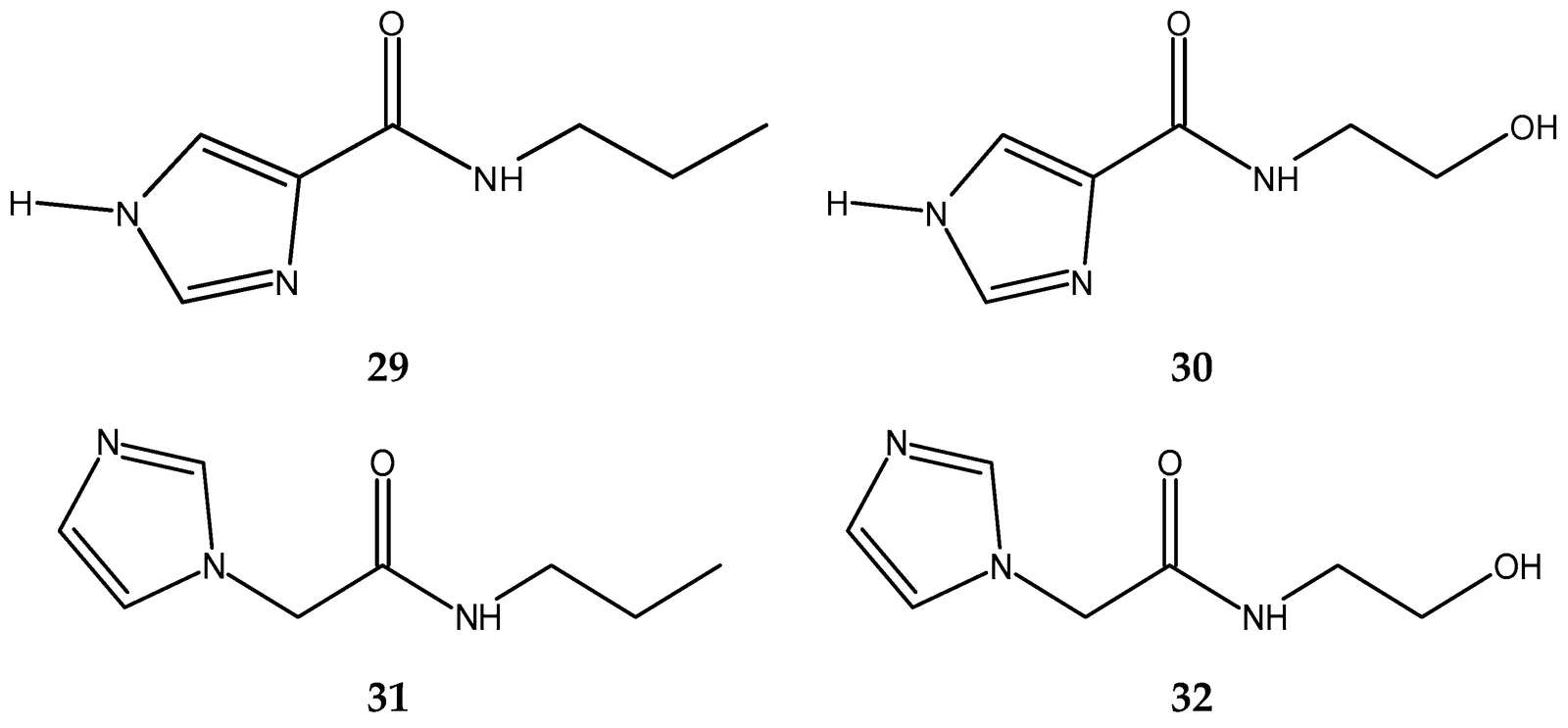
The leading derivatives of imidazole as potentially effective and environmentally and ecologically safe pesticides.

After filtering compounds with potential fungicidal activity, two promising compounds remain, namely, two alkyl-amides, **29** and **30**. For future environmentally safe herbicides, compounds that stand out are four alkyl-amides, **29**, **30**, **31**, and **32**, while compound **31** features as a leading insecticide. The structures of the leading compounds are presented in Figure 2.

**Figure 3:**
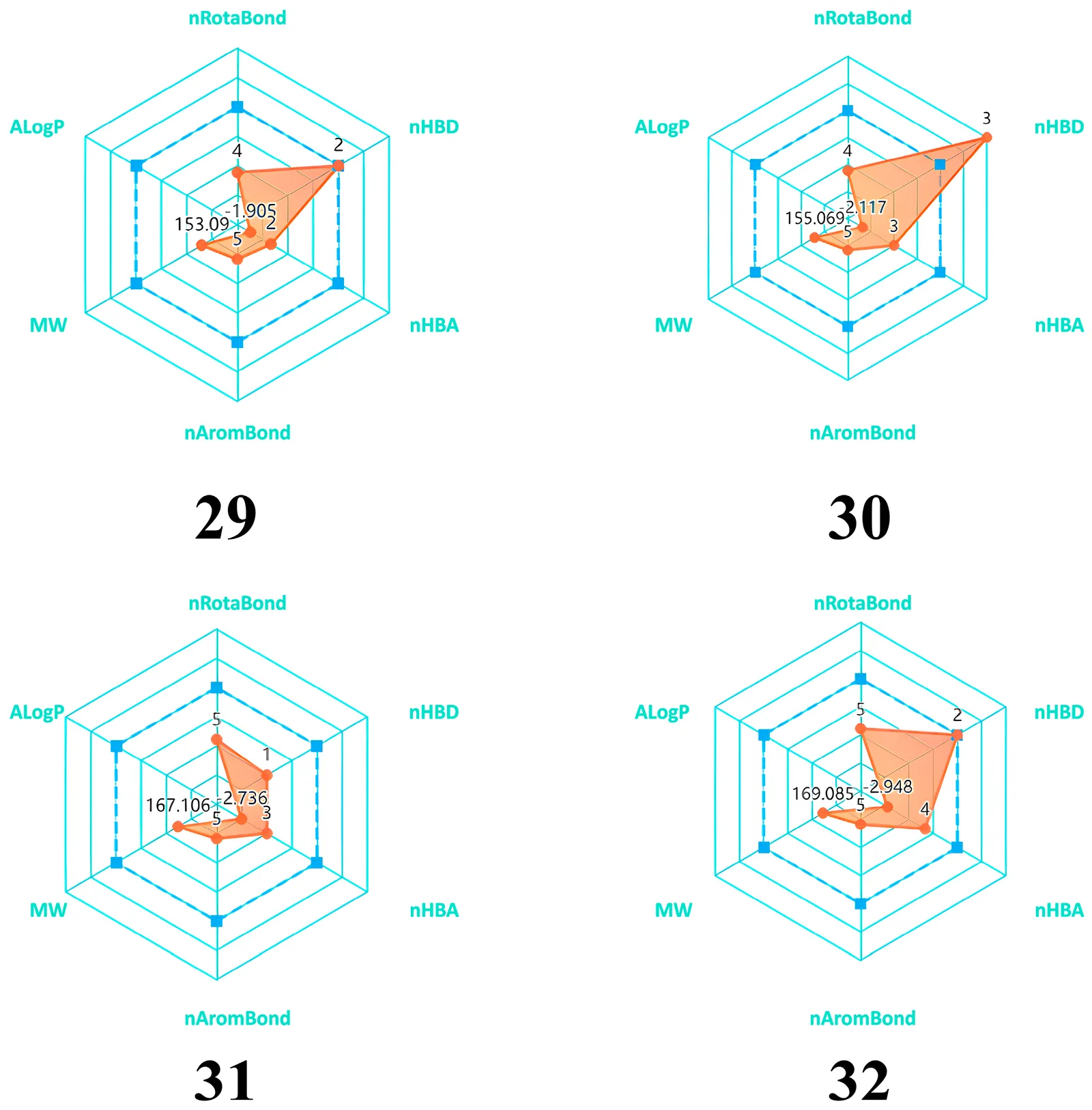
Radar plots of the qualitative evaluation of the pesticide-likeness parameters. nRotaBond (the number of rotatable bonds); nHBD (the number of H-bond donors); nHBA (the number of acceptors); nAromBond (the number of rotatable bonds); MW (molecular weight); Alog*P* (lipophilicity as Ghose–Crippen LogKow).

Radar plots of the qualitative evaluation of the pesticide-likeness parameters of the prominent compounds are presented in Figure 3.

According to the radar plots, all compounds satisfy the criteria of pesticide-likeness, which means that they have up to nine rotatable bonds, up to two H-bond donors, up to six H-bond acceptors, a molecular weight of up to 435, and Alog*P* up to 6. The exception is compound **30**, which has three H-bond donors, meaning that its polarity is too strong to penetrate cross-waxy cuticles on leaf surfaces or insect cell membranes [24].

A molecular docking study was conducted on targets related to each activity in order to identify the pesticides’ possible mechanism of action. In Table 3, the binding energies of the standout derivatives of imidazole and co-crystallized ligands are shown for the comparison.

To identify the derivatives’ possible mechanism of action against pathogenic fungi, a molecular docking study was performed on three enzymes responsible for fungal growth: proteinase K (PDB ID: 2PWB) [25]; demethylase (sterol 14α-demethylase (CYP51), PDB ID: 5EAH) [26]; and chitinase (PDB ID: 4TXE) [27].

To elucidate the possible mechanism of insecticidal activity, a docking study was performed on glutamate-gated chloride (CGluCl) channels as a target protein (pdb: 3RIF) [28] and on *Drosophila melanogaster* dopamine transporter (dDAT), which is structurally related to the GABA transporter isoforms (GATs) (PDB ID: 7WGT) [29].

For herbicidal activity, the carboxyltransferase domain of acetyl-coenzyme A carboxylase was chosen as the receptor (PDB ID: 3K8X) [30], while for antibacterial activity, peptide deformylase was selected (PDB ID: 1G2A) [31]. Crystal coordinates were provided from the Protein Data Bank (PDB, https://www.rcsb.org/). The results of molecular docking on enzymes related to antifungal activity are presented in Table 3. Root-Mean-Square Deviation (RMSD) is a measure of the average distance between the atomic positions of the re-docked ligand and its original crystallographic conformation. An RMSD value below 2 Å indicates the accuracy of the docking procedure.

Two featured compounds (**29** and **30**) for antifungal activity showed similar binding energies in complex with proteinase K and chitinase, but compound **30** had slightly higher energy with proteinase K (−87.69 kcal mol^−1^) compared with chitinase (−86.69 kcal mol^−1^). The reliability of the docking prediction was confirmed by the low RMSD. Compound **31**, which stood out as possibly the most active ecologically safe compound for insecticidal activity, had the highest binding energy (−72.09 kcal mol^−1^) in complex with the glutamate-gated chloride channel, while with the GABA transporter it had slightly higher binding affinity (−74.77 kcal mol^−1^). The accuracy of the docking results was confirmed by RMSD < 2. Among four preeminent compounds for herbicidal activity, compound **30** showed the highest binding energy (−56.00 kcal mol^−1^) in complex with carboxylase, and similarly stood out for antibacterial activity in complex with peptide deformylase (−83.63 kcal mol^−1^) (RMSD < 2).

**Figure 4:**
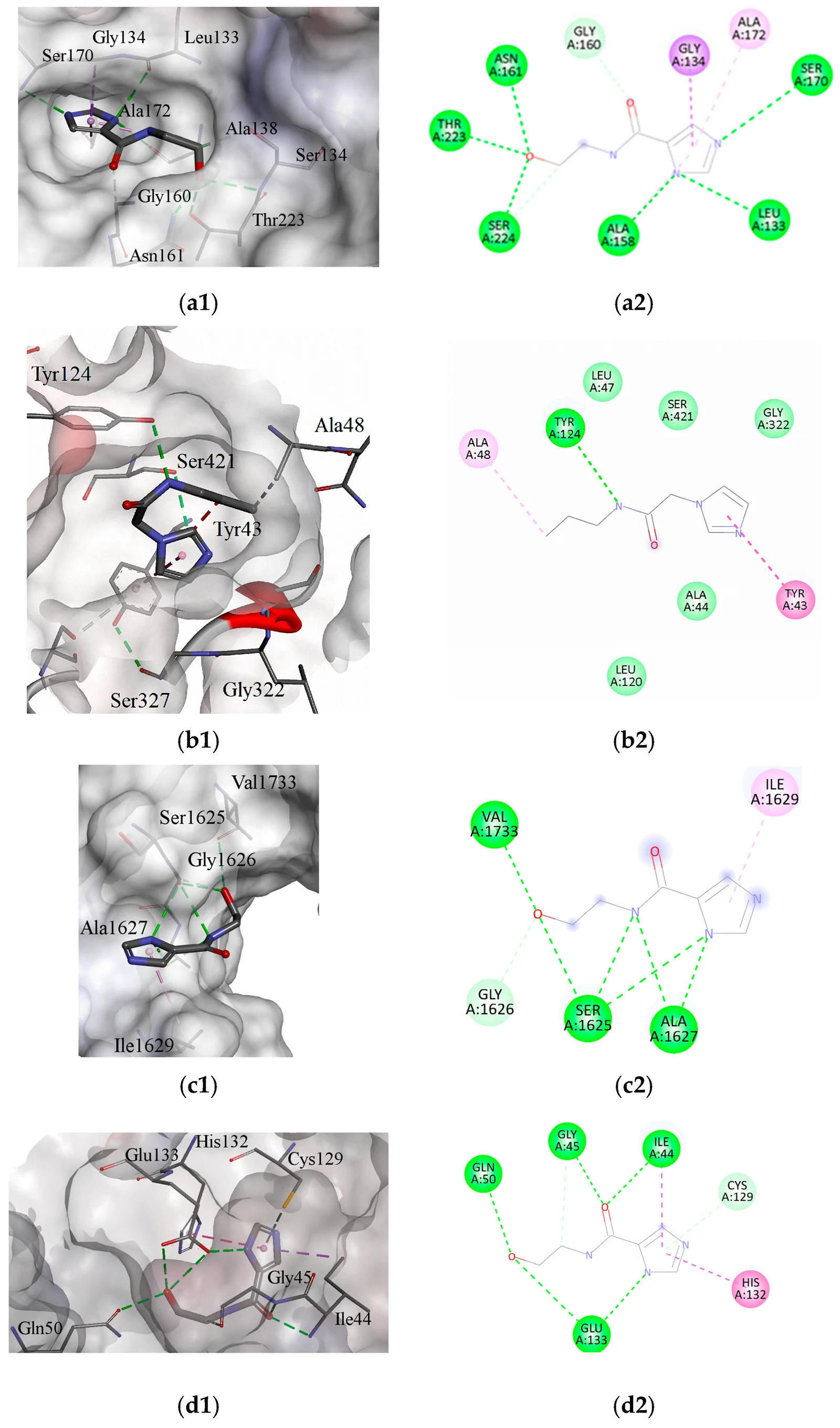
Docking pose (**1**) and main interactions (**2**) with amino acid residues of target protein and enzymes responsible for: (**a**) antifungal activity: (**a1**) proteinase K (PDB ID: 2PWB) with docked compound **30**; (**a2**) main interaction of compounds **30** with amino acid residues of proteinase K (PDB ID: 2PWB); (**b**) insecticidal activity: (**b1**) GABA transporter isoforms (PDB ID: 7WGT) with docked compound **31**; (**b2**) main interaction of compounds **31** with amino acid residues of GABA transporter isoforms (PDB ID: 7WGT); (**c**) herbicidal activity: (**c1**) carboxylase (PDB ID: 3K8X) with docked compound **30**; (**c2**) main interaction of compounds **30** with amino acid residues of carboxylase (PDB ID: 3K8X); (**d**) antibacterial activity: (**d1**) peptide deformylase: (PDB ID: 1G2A) with docked compound **30**; (**d2**) main interaction of compounds **30** with amino acid residues of peptide deformylase: (PDB ID: 1G2A). (green = conventional hydrogen bond; light green = van der Waals interactions; dark purple = π–σ interactions; light pink = π–π stacked; pink = π–alkyl).

Most of the co-crystallized reference ligands showed substantially more favorable binding energies than the studied compounds in molecular docking studies, which was expected. For example, co-crystallized ligand **38F** is an *Aspergillus fumigatus* plant-type chitinase inhibitor, which is an experimentally determined potent inhibitor of plant-type chitinase A1 [27]. Moreover, ivermectin (**IVM**) is an antiparasitic agent that binds to a GluCl channel common to invertebrate nerves and muscle cells and is used as an antiparasitic drug [28]. GABA transporter isoforms are a major inhibitory neurotransmitter and an important target for the study of insecticidal activity. The *D. melanogaster* dopamine transporter (dDAT) regulates dopamine signaling in the synaptic cleft. In the crystal structure (PDB ID: 7WGT), dDAT is in complex with a potent and selective inhibitor, namely, tiagabine analogs NO711 (PDB ID: **9BC**) [29]. Tepraloxydim (**B89**) is a commercial inhibitor of Acetyl-CoA carboxylase (ACCase), an important target enzyme for the development of new bleaching herbicides [30]. Therefore, it was expected that **B89** would achieve the highest binding energy. Actinonin (**BB2**) is a potent and selective inhibitor of peptide deformylase, an essential bacterial metalloenzyme, and also a natural antibiotic [31]. Figure 4 illustrates the positions of the docked compound **30** into the binding site of proteinase K, carboxylase, and deformylase, and compound **31** into the binding site of the glutamate-gated chloride channel, along with 2D diagrams of the main interactions with amino acid residues.

## 3. Discussion

Both adamantane and alkyl derivatives of imidazole show potential as pesticides, but adamantane has the highest affinity for becoming an antifungal agent. The adamantane cage is an important moiety for diverse biological properties. Thus, adamantane-containing thiazole compounds have been shown to have *in vitro* antibacterial, antifungal, and antiproliferative activity towards five human tumor cell lines and inhibitory activity against orthopoxviruses [32,33,34]. In pest management, the derivatives of adamantane were proven to have inhibitory activity against detoxification enzymes in pest insects, such as glucosinolate sulfatases, which affects their ability to break down plant defense toxins [35]. In addition, supramolecular complexes of adamantane-functionalized 1,3,4-oxadiazoles and β-cyclodextrin are bioactive against bacterial phytopathogens *Xanthomonas oryzae* pv. *oryzae* (*Xoo*), *Xanthomonas axonopodis* pv. *citri* (*Xac*), and *Pseudomonas syringae* pv. *actinidiae* (*Psa*) [36].

The criterion of biodegradability excluded numerous potentially active compounds from the initial set, leaving only alkyl derivatives of imidazole. Chemicals can break down through hydrolysis, biodegradation, direct or indirect photolysis, and redox reactions, which can result in their release into the environment and environmental risk events. Adamantane’s robust cage-like structure makes it resistant to natural breakdown in the environment, leading to long-term persistence and its characterization as not readily biodegradable [23]. Moreover, for aquatic organisms, the most toxic compounds are highly lipophilic derivatives, especially with the adamantane moiety.

Triazoles are among the most common systemic fungicides used in plant disease control. Coumarin-1,2,4-triazole hybrids effectively inhibited the mycelial growth of plant pathogenic fungi *Sclerotinia sclerotiorum* and *Fusarium oxysporum* [37]. However, studies demonstrated that triazole fungicides exert development toxicity in zebrafish, causing disruption to their energy metabolism [38]. Although carboxamide derivatives possess a broad spectrum of antifungal activities, allowing them to act as succinate dehydrogenase inhibitors [39], due to their pronounced hydrophilicity, they have a great probability for aquatic toxicity, especially in combination with the chlorophenyl moiety. Acetamido-2-ylidene derivatives of imidazole have shown great potential as antibacterial agents against *Escherichia coli* [40]. Imazalil is the active substance of fungicides, which means that it protects plants against fungal diseases. Only a few toxicological studies have assessed the potential environmental effect of imazalil and its impact on organisms. This pesticide can cause morphological anomalies and locomotion changes in fish (*Danio rerio*) but did not cause immediate earthworm *E. andrei* death after exposure [17]. Prochloraz is a fungicide active against a wide range of diseases affecting cereals, field crops, fruit, and many other crops, but it is not approved by the EC since it inhibits fetal steroidogenesis [41].

Increased toxicity is related to the presence of multiple rings, both aliphatic and aromatic (three rings, for example); this is explained by the greater lipophilicity of these large compounds with many carbon atoms [42]. Therefore, Alog*P* is one of the descriptors included in QSAR models for the prediction of toxicity on fish, fathead minnow, and aquatic invertebrates *D. magna*, alongside BCF.

Four alkyl derivatives of imidazoles featured as promising environmentally and ecologically safe pesticides. Alkyl derivatives of imidazole are extremely adaptable heterocyclic scaffolds that function as the fundamental building blocks of contemporary agricultural fungicides, bactericides, and insecticides. For targeted crop protection and specialized pest control, their structures can be modified by changing the lengths of the alkyl chains or the nitrogen atoms in the imidazole ring [13].

Our molecular docking studies indicated the possible mechanisms of prominent imidazole derivatives. A molecular docking study for antifungal activity was performed on the enzymes responsible for plant cell wall-degradation, i.e., proteinase K (PDB ID: 2PWB), and fungal growth, namely, 14α-demethylase (PDB ID: 5EAH) and chitinase (PDB ID: 4TXE). These target enzymes have also been used in our previous studies on the antifungal activities of coumarins [43]; coumarinyl Schiff bases [44]; fluoro-substituted pyrazoles [45]; and coumarin-1,2,4-triazole hybrid (CTH) compounds [37].

The antifungal activity of carboxamide derivatives of imidazole (**30**) could be related to the inhibition of proteinase K. Proteinase K is a fungal proteolytic enzyme that is involved in the degradation of the host extracellular matrix during the penetration and colonization of phytopathogenic fungi. A serine proteinase, found in the phytopathogenic fungus *Fusarium solani* f.sp. *eumartii*, hydrolyzes specific polypeptides of potato intercellular washing fluids and cell walls [25]. The binding site was defined according to the crystal structure of the coumarin complex with proteinase K (PDB ID: 2PWB). The active site of proteinase K consists of the catalytic triad Asp39-His69-Ser224, and the substrate recognition site is formed by Gly100-Tyr104 and Ser132-Gly136 [46].

The possible mechanism of inhibition of compound **30**, revealed by molecular docking, is based on the generation of H-bonds between the 2-hydroxyl group and Asn161, Thr223, and Ser224. In addition, the nitrogen atoms from the imidazole core generate a H-bond with Leu133, Ala158, and Ser170. The aromatic ring of the imidazole core produces π–π interactions with Gly134 and π–π stacked interactions with Ala172 (Figure 4(a1,a2)). In our previous study, a molecular docking study demonstrated that fluorinated pyrazole aldehydes could be better inhibitors of proteinase K than coumarin as a standard inhibitor [45]. That study revealed a lower binding affinity of coumarin compared to the studied pyrazole aldehydes, which is in agreement with the results of the present molecular docking. Coumarin created three hydrogen bonds between the 2-oxo group and Asn161, Ser224, and Thr223. Coumarin’s fewer interactions could be explained by the lack of nitrogen atoms in its structure compared to the derivatives of imidazole and fluorinated pyrazole aldehydes.

The selection of protein targets for insecticidal activity was based on our previously published paper on the insecticidal activity of CTH compounds against *D. melanogaster* [47]. Since these compounds showed great insecticidal potential against *D. melanogaster*, to elucidate their possible mechanism of action, a docking study was performed on glutamate-gated chloride (CGluCl) channels as a target protein (PDB ID: 3RIF) into the binding site of the antiparasitic drug ivermectin (PDB ID: IVM), as well as on *D. melanogaster* dopamine transporter (dDAT) (PDB ID: 7WGT). The molecular docking study implied that they are probably acting as glutamate-gated chloride channel inhibitors. Dopamine transporter (DAT) facilitates dopamine reuptake from the extracellular space utilizing the Na^+^ gradient and thus terminates neurotransmission [48]. Certain insecticides, such as the pyrethroid insecticide permethrin, directly target or alter DAT, acting as neurotoxins [49]. Compound **31** is fitted into the binding site of inhibitor NO-711 (PDB ID: 9BC), generating the H-bond through the amide N atom with Tyr124. The imidazole ring produces van der Waals interactions with Tyr43 and Ser327, as well as a π–alkyl interaction between the propyl chain and Ala48 (Figure 4(b1,b2)). NO711 also interacts with the hydroxyl group of Tyr124, while the phenyl group of NO711 forms a network of aromatic and hydrophobic interactions with Phe318, Phe319, and a displaced Leu325 [29].

Herbicidal activity was related to the inhibition of carboxyltransferase (CT) activity of acetyl-CoA carboxylase (ACC), a crucial enzyme for the metabolism of fatty acids [30]. The co-crystalized ligand in the complex PDB ID: 3K8X is tepraloxydim, a commercial herbicide which kills sensitive plants by inhibiting the carboxyltransferase (CT) activity of ACC. Compound **30** was bound into the bond in the active site region of CT, according to the binding mode of the herbicide tepraloxydim. Similar to tepraloxydim, which is hydrogen-bonded with Ala1627, compound **30** binds with the same residue with two H-bonds, one by the pyridine-type N atom of imidazole and another with the carboxamide N atom. The hydroxyl group is very important as it creates an H-bond with Val1733 and Ser1625. Ser1625 is also hydrogen-bonded with a pyridine-type N atom. The imidazole ring produces π-alkyl interactions with Ile1629 (Figure 4(c1,c2)).

Peptide deformylase (PDF) is a bacterial metalloenzyme that participates in bacterial protein biosynthesis through the deformylation of the terminal methionine residue. Inhibitors of PDF prevent that step and thus enable bacterial growth [31]. The co-crystalized ligand in the complex PDB ID: 1G2A is actinonin, a potent and selective inhibitor of PDF and a natural antibiotic [31]. Compound **30** was docked into the actinonin binding site of PDF (PDB ID: 1G2A) creating H-bonds among the hydroxyl group and Gln50 and Glu133; it also bonded a carboxamide oxygen atom with Gly45 and Ile44, and a pyrrole-type N atom of imidazole ring with Glu133. In addition, π electrons from the imidazole ring produce a π–σ interaction with His132 (Figure 4(d1,d2)). Inhibitor actinonin creates more H-bonds than compound **30** as it has more H-bond donor atoms. Actinonin creates H-bonds between Ile44 and an oxygen atom from the carbonyl group, as well as between a main-chain carbonyl oxygen atom and NH with Gly89. It also creates a H-bond between the terminal alcohol group and the carbonyl oxygen of Glu87, and between hydroxamate or the *N*-formyl-hydroxylamine and the side chains of Glu133 and Gln50, and the main-chain NH of Leu91 [31].

### Limitations of the Study

Although the purpose of this study was the *in silico* ecotoxicological assessment of designed, non-synthesized imidazole derivatives for a rational approach to designing highly potent agents for plant protection, it has several limitations, and all conclusions are preliminary and require experimental confirmation.

One of the limitations is that the estimated pesticide similarity and environmental risk properties calculated using the Chem-FREE web platform should be considered with caution since the checking chemical in the applicability domain is not possible. However, researchers have indicated that these limitations may refer to carbohydrates and biomolecules [50]. Moreover, the ecotoxicological effects of potential imidazole derivatives calculated by VEGA mostly have a low reliability of prediction, defined according to the applicability domain of the QSAR models, and should be interpreted with caution.

The results of the molecular docking represent hypothetical binding modes, and in the final phase of the investigation, they must be confirmed experimentally.

## 4. Materials and Methods

The names of the 32 imidazole derivatives are presented in Table 4. The chemical structures of these compounds were converted into SMILES notations, which served as input into the platforms for the calculation of their pesticide-likeness and eco-environmental risk properties. The structures and SMILES notations of the compounds are listed in the Supplementary Materials, Table S1.

### 4.1. Pesticide-Likeness Properties and Environmental Risk Evaluation

The prediction of the pesticide-likeness properties and environmental risk assessment was performed with the aid of the ChemFREE platform, which is freely available at https://chemfree.agroda.cn/chemfree/#/Home (accessed on 5 July 2026) ChemFREE web integrates QSAR models that allow the quantitative evaluation of the probability of chemicals becoming pesticides (fungicide, insecticide, or herbicide), as well as the biodegradability and persistence of chemicals in various environments (water, soil, sediment, and air). The quantitative evaluation of pesticide-likeness was performed using PaDEL-Descriptor v1.0 software. Models were evaluated by the AUC (area under the receiver operating characteristic (ROC) curve) parameter, which was in the range 0.740–0.870. Biodegradability was estimated by a rule-based method with an accuracy (ACC) of 0.922. Degradation half-life was estimated using graph convolutional network (GACNN) and graph attention convolutional neural network (GACNN) models, with *R*^2^ values of 0.960 (for soil), 0.84 (for sediment), 0.81 (for air), and 0.89 (for water) [50].

Pesticide-likeness is expressed by the desirability index (RDL), which represents the probability of chemicals becoming pesticides. The higher the RDL scores, the more similar the compound is to a pesticide. The persistence in water, soil, and sediment is indicated by degradation half-life (HL), which describes the ability of chemicals to remain unchanged in the environment. The HL thresholds are set at 40 and 60 days for water and soil and 120 and 180 for sediment. Chemicals are categorized as air-non-persistent if HL > 2 days.

The biodegradability prediction model classified compounds as readily biodegradable if they can degrade by 60% within 28 days or not readily biodegradable if they do not. The similarity of the compounds to pesticides is visually presented as a radar plot that includes several pesticide-likeness parameters: flexibility (the number of rotatable bonds, ROB); polarity (H-bond donors, HBD, and H-bond acceptors, HBA); photostability (aromatic bonds, ARBs); size (molecular weight, MW); and lipophilicity (Ghose–Crippen LogKow, Alog*P*).

The VEGA platform at https://www.vegahub.eu/about-vegahub/ (accessed on 6 July 2026) allows access to a series of QSAR (quantitative structure–activity relationship) models for the prediction of environmental hazard properties. It uses QSAR models that have been optimized in accordance with the REACH requirements [7]. For the calculation of ecotoxicology properties, we used the following QSAR models: (A) fish acute toxicity model (NIC), LC50 [log(1/(mmol L^−1^))]; (B) short-term toxicity to fish fathead minnow (*Pimephales promelas*), LC50 96 h (EPA) [−log(mol L^−1^)]; (C) short-term toxicity to aquatic invertebrates (*Daphnia magna*), LC50 48 h (EPA)-prediction [−log(mol L^−1^)]; (D) algae classification toxicity model (ProtoQSAR/Combase) according to a growth inhibition test; binary classification: toxic (ErC50 < 10 mg L^−1^) or NON-toxic (ErC50 ≥ 10 mg L^−1^); (E) algae acute toxicity model (ProtoQSAR-Combase), EC50 [mg L^−1^]; (F) bee acute toxicity model (kNN/IRFMN), median lethal dose (LD50) after 48 h [μg bee^−1^]; (G) earthworm toxicity (CONCERT) [−log mg kg^−1^]; (H) sludge (EC50) toxicity model (ProtoQSAR-Combase) [mg L^−1^]; (I) bioconcentration factor (BCF) model (Maylan), the concentration of test substance in the fish divided by the concentration of the chemical in the surrounding medium at steady state [log(L kg^−1^)]. The lipophilicity of compounds was calculated by the online ALOGPS 2.1 program [51]. The reliability of prediction, defined according to the applicability domain of the model, is indicated as good, moderate, or low. The QSAR Model Reporting Format (QMRF) of the VEGA models is given at https://www.vegahub.eu/portfolio-item/vega-qsar-models-qrmf/ (accessed on 6 July 2026).

### 4.2. Molecular Docking

The molecular docking study was preceded by the preparation of the ligands (molecules) and receptors (enzymes and proteins). The structures of the compounds were optimized via Spartan ’08 v1.2.0. (Wavefunction, Inc.; Irvine, CA, USA, 2009) using the molecular mechanics force field (MM+) [52] and reoptimized by the semiempirical quantum-mechanical method PM3 [53] using the Polak–Ribiere algorithm until the root mean square gradient (RMS) was 0.001 kcal mol^−1^ per Å.

To suggest the possible mechanism of action of derivatives against pathogenic fungi, a molecular docking study was performed on three enzymes responsible for fungal growth: proteinase K (PDB ID: 2PWB) [25]; demethylase (sterol 14α-demethylase (CYP51), PDB ID: 5EAH) [26]; and chitinase (PDB ID: 4TXE) [27].

For the insecticidal activity, a docking study was performed on glutamate-gated chloride (CGluCl) channels as a target protein (PDB ID: 3RIF) [28] and on *D. melanogaster* dopamine transporter (dDAT) which is structurally related to the GABA transporter isoforms (GATs) (PDB ID: 7WGT) [29].

For herbicidal activity, the carboxyltransferase domain of acetyl-coenzyme A carboxylase was chosen as the receptor (PDB ID: 3K8X) [30], while for antibacterial activity peptide deformylase was selected (PDB ID: 1G2A) [31]. Crystal coordinates were provided from the Protein Data Bank (PDB, https://www.rcsb.org/).

Molecular docking was performed with iGEMDOCK v2.1 (BioXGEM, Hsinchu, Taiwan) using a generic evolutionary method (GA). The GA parameters were set as follows: population size 200; generations 70; number of poses 3; and binding site radius 8 Å. The output of molecular docking is energy-based scoring function (kcal mol^−1^), integrating an empirical-based energy function, binding-site pharmacological interactions, and ligand preferences:

*E*_total_ = *E*_bind_ + *E*_pharma_ + *E*_ligpre_ (1)

where *E*_bind_ is the empirical binding energy; *E*_pharma_ is the energy of binding site pharmacophores; and *E*_ligpre_ is a penalty value if a ligand does not satisfy the ligand preferences [54].

Receptor–ligand interactions were visualized with BIOVIA Discovery Studio Visualizer 4.5 (Dassault Systèmes, San Diego, CA, USA).

To validate the molecular docking methodology, as well as its reliability and ability for the accurate prediction of ligand–receptor interactions, a re-docking process was employed. Re-docking involves repositioning a ligand back into the original binding site of its receptor, using its known crystallographic structure as a reference. The obtained Root-Mean-Square Deviation (RMSD) value evaluates the accuracy of the re-docking process. Re-docking was performed with iGEMDOCK, and RMSD was calculated using BIOVIA Discovery Studio Visualizer 4.5 software. The RMSD of the docked co-crystallized ligand was within the reliable range of 2 Å.

## 5. Conclusions

This study represents an initial stage in the rational development of plant protection products based on a chemoinformatic approach that allows preliminary testing of molecules for toxicity and environmental effects. A set of designed imidazole derivatives (adamantane-, alkyl-, and triazole-amides, esters, carbamates, and ketones) were filtered based on estimated pesticide-likeness, environmental risk properties, and ecotoxicological effects. Four alkyl-amides stood out as promising agents for sustainable crop protection. A molecular docking study suggested a possible mode of action for antifungal, antibacterial, herbicidal, and insecticidal activity and implied structural features important for binding to a specific receptor.

The next step of this research will involve “green” synthesis and bioassays on pathogenic and beneficial organisms.

## Figures and Tables

**Table 1:** Pesticide-likeness and environmental risk properties of imidazole derivatives.

Compound	Fungicide-Likeness *	Herbicide-Likeness *	Insecticide-Likeness *	Biodegradable	Degradation Half-Life			
					Air	Soil	Water	Sediment
**1**	0.838	0.951	0.887	Non	10.759	22.918	22.501	13
**2**	0.84	1.03	0.914	Non	4.483	25.836	4.198	13
**3**	0.838	0.951	0.887	Non	7.704	29.18	4.198	13
**4**	0.822	0.985	0.985	Non	18.49	22.918	22.501	13
**5**	1.003	0.879	0	Non	46.663	22.918	22.501	13
**6**	1.228	0.891	0	Non	1.313	22.918	4.198	13
**7**	1.205	0.923	0	Non	19.442	22.918	4.198	13
**8**	0.966	1.138	0.95	Non	1.787	25.836	7.075	13
**9**	1	1.176	1.053	Non	4.289	22.918	7.075	13
**10**	0.873	0.936	0.996	Non	4.821	22.918	7.075	13
**11**	0.875	1.014	1.027	Non	2.009	22.918	7.075	13
**12**	0.994	1.235	0.922	Non	0.046	22.942	2.305	41
**13**	1	1.601	1.227	Non	0.018	22.942	7.063	41
**14**	0.873	1.426	1.102	Non	0.02	22.942	7.063	41
**15**	0.975	1.279	1.024	Non	0.04	22.942	2.305	71
**16**	1.09	1.482	0.902	Non	0.909	33.755	7.075	13
**17**	0.927	1.156	0.846	Non	2.189	25.836	7.075	13
**18**	1.247	1.345	0.926	Non	0.00815	10.672	7.063	228
**19**	0.684	0.932	0.61	Non	0.01	40.934	6.197	41
**20**	0.749	0.934	0.684	Yes	0.011	22.942	6.197	41
**21**	0.585	0.68	0.553	Non	0.003	73.558	8.465	23
**22**	0.585	0.68	0.553	Non	0.0422	73.558	8.465	23
**23**	0.621	0.726	0.517	Non	0.00233	73.558	8.465	23
**24**	0.621	0.726	0.517	Non	0.00332	73.558	8.465	23
**25**	0.864	1.007	0.845	Non	0.00227	40.934	8.465	41
**26**	0.959	0.997	0.801	Yes	0.0058	40.934	8.465	41
**27**	1.275	1.487	1.056	Yes	0.184	12.669	6.197	228
**28**	1.249	1.568	0.961	Non	0.177	12.699	6.197	228
**29**	1.042	1.485	0.97	Yes	0.049	7.068	6.197	73
**30**	0.696	1.099	0.872	Yes	0.035	4.954	6.197	41
**31**	0.965	1.232	1.114	Yes	0.051	7.068	6.197	73
**32**	0.881	1.078	0.92	Yes	0.026	22.942	6.197	41

* Desirability index (RDL), which represents the probability of chemicals becoming pesticides.

**Table 2:** Predicted ecotoxicological effects of potential imidazole derivatives. Reliability of prediction, defined according to the applicability domain of the model, is indicated as follows: G (good); M (moderate); L (low).

Comp.	Fish Acute Tox. ^1^	Fathead Minnow ^2^	*Daphnia magna* ^3^	Algae Tox. ^4^	Algae Acute ^5^	Bees ^6^	Earthworm ^7^	Sludge ^8^	BCF ^9^	Alog*P* ^10^
	LC50 [log(1/ (mmol L^−1^))]	LC50 [−log(mol L^−1^)]			EC50 [mg L^−1^]		[−log mg kg^−1^]	EC50 [mg L^−1^]	[log(L kg^−1^)]	
**1**	4.49 L	3.73 L	5.5 L	+L	0.188 L	Low M	−3.246 L	66.58 L	2.51 L	1.77 G
**2**	1.93 L	3.80 L	5.19 L	+L	0.183 L	Low M	−2.766 L	47.15	2.18 L	2.83 G
**3**	4.49 L	3.98 L	5.23 L	+L	0.160 L	Low M	−2.965 L	41.90	2.54 L	3.18 G
**4**	4.49 L	3.91 L	5.53 L	+L	0.165 L	Low M	−3.454 L	58.34	2.87 L	3.44 G
**5**	4.49 L	3.97 L	5.12	+L	0.142 L	Low M	−3.558 L	54.66	3.23 L	−0.58 G
**6**	4.49 L	3.87 L	4.78 L	+L	0.169 L	Low M	−2.853 L	45.61	2.54 L	−0.94 G
**7**	4.49 L	4.04 L	4.83 L	+L	0.138 L	Low M	−3.083 L	40.24	2.90 L	−0.84 G
**8**	2.10 L	4.18 L	4.76 L	+L	0.097 L	Low L	−1.227 L	29.51	2.56 L	3.03 G
**9**	1.93 L	4.07 L	5.10 L	+L	0.099 L	Low L	−1.730 L	57.96	2.89 L	3.43 G
**10**	4.49 L	4.12 L	5.12 L	+L	0.431 L	Low L	−3.148 L	46.92	3.21 L	3.64 G
**11**	2.49 L	4.21 L	4.78 L	+L	0.419 L	Mod L	−2.694 L	35.96	2.89 L	3.36 G
**12**	1.43 L	2.54 L	3.32 L	+L	0.482 L	Mod L	−2.164 L	39.04	0.46 L	0.56 G
**13**	0.71 L	2.98 L	2.88 L	+L	0.230 L	Mod L	−0.999 L	17.76	0.50 L	0.54 G
**14**	0.45 L	2.96 L	2.88 L	+L	1.090 L	Mod L	−2.113 L	24.41	0.50 L	0.72 G
**15**	1.43 L	2.72 L	3.45 L	+L	0.429 L	Mod M	−2.366 L	38.30	0.82 L	0.87 G
**16**	2.10 L	4.21 L	5.38 L	+L	0.475 L	Low M	−2.774 L	40.49	2.24 L	2.89 G
**17**	2.10 L	4.12 L	5.71 L	+L	0.488 L	Low M	−3.195 L	47.49	2.56 L	3.13 G
**18**	0.71 L	3.09 L	3.46 L	+L	2.940 L	Mod L	−1.948 L	12.43	0.50 L	0.50 G
**19**	1.84 L	2.37 L	2.52 L	−L	131.92 L	Low L	−0.112 L	59.03	0.50 L	−0.45 G
**20**	1.18 L	2.19 L	1.95 L	−L	130.33 L	Low L	−2.226 L	63.49	0.50 L	−0.87 G
**21**	1.93 L	3.72 L	2.72 L	+L	0.020 L	Low L	−1.957 L	52.00	0.50 L	1.37 G
**22**	2.83 L	3.71 L	2.72 L	+L	0.021 L	Low L	−1.780 L	61.37	0.50 L	1.38 G
**23**	1.93 L	3.88 L	3.00 L	+L	0.021 L	Low L	−1.988 L	52.38	0.50 L	1.32 G
**24**	1.93 L	3.87 L	3.00 L	+L	0.022 L	Low L	−1.796 L	64.28	0.50 L	1.33 G
**25**	0.25 L	2.42 L	1.72 L	−L	148.780 L	Low L	−0.168 L	46.24	0.50 L	−0.45 G
**26**	1.62 L	2.24 L	1.00 L	−L	144.480 L	NP	−0.445 L	49.78	0.50 L	−0.91 G
**27**	0.69 L	2.74 L	3.47 L	−L	190.550 L	NP	−0.906 L	71.84	0.50 L	−0.34 G
**28**	0.69 L	2.88 L	3.58 L	−L	192.520 L	Low G	−0.426 L	79.90	0.50 L	−0.21 G
**29**	0.71 L	2.67 L	3.51 L	−M	192.520 L	Low M	−0.775 L	93.23	0.50 L	0.32 G
**30**	1.21 L	1.75 L	2.35 L	−L	43.340 L	Low M	−2.062 L	79.31	0.50 L	−0.96 G
**31**	0.71 L	2.54 L	3.37 L	−L	190.550 L	Mod M	−1.351 L	68.51	0.50 L	0.23 G
**32**	1.59 L	1.54 L	2.57 L	−L	248.210 L	Low M	−2.649 L	87.46	0.50 L	−1.28 G
Cutoff values	highly toxic LC50 > 4	highly toxic LC50 > 4	highly toxic LC50 > 4		high-concern EC50 < 1		toxic ≤ −2	toxic ≤ 50	toxic > 3	

^1^ Fish acute toxicity. ^2^ Short-term toxicity to fish (fathead minnow). ^3^ Short-term toxicity to *Daphnia magna*. ^4^ Algae classification toxicity: (+) toxic; (−) non-toxic. ^5^ Algae acute toxicity. ^6^ Bee acute toxicity: Low (>100 µg/bee); Mod (1–100 µg/bee). ^7^ Earthworm toxicity. ^8^ Sludge toxicity. ^9^ The bioconcentration factor (BCF) model. ^10^ Alog*P* = lipophilicity; NP = not predicted.

**Table 3:** Energy-based scoring function (kcal mol^−1^) of interactions of the best docked poses of four the leading candidates in complex with proteinase K; sterol 14α-demethylase; chitinase; glutamate-gated chloride (CGluCl) channels; GABA transporter isoforms; carboxyltransferase domain of acetyl-coenzyme A carboxylase; and peptide deformylase. The Root-Mean-Square Deviation (RMSD) values of the re-docking process are in parentheses.

Effect:	Antifungal			Insecticidal		Herbicidal	Antibacterial
Target:	proteinase K	14α-demethylase	chitinase	glutamate-gated chloride channel	GABA transporter isoforms	carboxylase	peptide deformylase
PDB ID:	2PWB	5EAH	4TXE	3RIF	7WGT	3K8X	1G2A
Co-crystalized ligand	coumarin	triazole	plant-type chitinase inhibitor	ivermectin	NO-711	tepraloxydim	actinonin
PDB ID:	COU	5LWT	38F	IVM	9BC	B89	BB2
Co-crystalized ligand	−72.30	−57.13	−124.11	−158.08	−123.177	−74.31	−131.68
**29**	−78.04 (1.97 Å)	−58.15 (9.22 Å)	−77.25 (1.09 Å)	−74.56 (0.21 Å)	−76.91 (0.11 Å)	−58.36 (0.49 Å)	−57.99 (1.73 Å)
**30**	−87.69 (0.46 Å)	−67.48 (4.64 Å)	−86.23 (0.62 Å)	−71.48 (4.71 Å)	−79.38 (0.51 Å)	−59.42 (1.07 Å)	−83.63 (0.66 Å)
**31**	−91.28 (0.48 Å)	−61.24 (0.72 Å)	−85.50 (0.32 Å)	−72.09 (1.55 Å)	−74.77 (1.44 Å)	−56.00 (0.54 Å)	−77.65 (4.82 Å)
**32**	−91.08 (0.55 Å)	−65.54 (9.07 Å)	−92.02 (0.88 Å)	−74.00 (2.03 Å)	−85.16 (1.96 Å)	−57.18 (0.48 Å)	−92.11 (0.66 Å)

**Table 4:** Names of studied derivatives of imidazole.

Compound	Name
**1**	*N*-(3-(1*H*-imidazol-1-yl)propyl)-2-((3*r*,5*r*,7*r*)-adamantan-1-yl)acetamide
**2**	(3*r*,5*r*,7*r*)-*N*-(3-(1*H*-imidazol-1-yl)propyl)adamantane-1-carboxamide
**3**	(3*r*,5*r*,7*r*)-*N*-(3-(2-methyl-1*H*-imidazol-1-yl)propyl)adamantane-1-carboxamide
**4**	2-((3*r*,5*r*,7*r*)-adamantan-1-yl)-*N*-(3-(2-methyl-1*H*-imidazol-1-yl)propyl)acetamide
**5**	1-(3-(2-((3*r*,5*r*,7*r*)-adamantan-1-yl)acetamido)propyl)-2,3-dimethyl-1*H*-imidazol-3-ium iodide
**6**	1-(3-((3*r*,5*r*,7*r*)-adamantane-1-carboxamido)propyl)-3-methyl-1*H*-imidazol-3-ium iodide
**7**	1-(3-((3*r*,5*r*,7*r*)-adamantane-1-carboxamido)propyl)-2,3-dimethyl-1*H*-imidazol-3-ium iodide
**8**	2-(1*H*-imidazol-1-yl)ethyl (3*r*,5*r*,7*r*)-adamantane-1-carboxylate
**9**	2-(1*H*-imidazol-1-yl)ethyl 2-((3*r*,5*r*,7*r*)-adamantan-1-yl)acetate
**10**	3-(1*H*-imidazol-1-yl)propyl 2-(adamantan-1-yl)acetate
**11**	3-(1*H*-imidazol-1-yl)propyl (3*r*,5*r*,7*r*)-adamantane-1-carboxylate
**12**	ethyl (3-(1*H*-imidazol-1-yl)propyl)carbamate
**13**	2-(1*H*-imidazol-1-yl)ethyl ethyl carbonate
**14**	3-(1*H*-imidazol-1-yl)propyl ethyl carbonate
**15**	ethyl (3-(2-methyl-1*H*-imidazol-1-yl)propyl)carbamate
**16**	(1*H*-imidazol-5-yl)methyl (3*r*,5*r*,7*r*)-adamantane-1-carboxylate
**17**	(1*H*-imidazol-5-yl)methyl 2-((3*r*,5*r*,7*r*)-adamantan-1-yl)acetate
**18**	(1*H*-imidazol-5-yl)methyl ethyl carbonate
**19**	*N*-((1λ^2^,2,3-triazol-4-yl)methyl)-1*H*-imidazole-4-carboxamide
**20**	*N*-((1*H*-1,2,3-triazol-4-yl)methyl)-2-(1*H*-imidazol-1-yl)acetamide
**21**	*N*-((1-(4-chlorophenyl)-1*H*-1,2,3-triazol-4-yl)methyl)-2-(1*H*-imidazol-1-yl)acetamide
**22**	*N*-((1-(3-chlorophenyl)-1*H*-1,2,3-triazol-4-yl)methyl)-2-(1*H*-imidazol-1-yl)acetamide
**23**	*N*-((1-(4-chlorophenyl)-1*H*-1,2,3-triazol-4-yl)methyl)-1*H*-imidazole-4-carboxamide
**24**	*N*-((1-(3-chlorophenyl)-1*H*-1,2,3-triazol-4-yl)methyl)-1*H*-imidazole-4-carboxamide
**25**	(1*H*-imidazol-4-yl)(1*H*-1,2,4-triazol-1-yl)methanone
**26**	2-(1*H*-imidazol-1-yl)-1-(1*H*-1,2,4-triazol-1-yl)ethan-1-one
**27**	2-(1*H*-imidazol-1-yl)-*N*-(prop-2-yn-1-yl)acetamide
**28**	*N*-(prop-2-yn-1-yl)-1*H*-imidazole-4-carboxamide
**29**	*N*-propyl-1*H*-imidazole-4-carboxamide
**30**	*N*-(2-hydroxyethyl)-1*H*-imidazole-4-carboxamide
**31**	2-(1*H*-imidazol-1-yl)-*N*-propylacetamide
**32**	*N*-(2-hydroxyethyl)-2-(1*H*-imidazol-1-yl)acetamide

## Data Availability

Data are contained within the article.

## Supplementary Materials

The following supporting information can be downloaded at https://www.mdpi.com/article/10.3390/molecules31183166/s1, Table S1. Structures and SMILES notations of designed imidazole derivatives.
