# Supplementary material for: QSAR-Based Ecotoxicological Assessment of Novel Imidazole Derivatives for Sustainable Crop Protection
Source: Molecules. 2026 Sep 9;31(18):3166. doi: 10.3390/molecules31183166 (PMC13609507; doi:10.3390/molecules31183166)
Supplement: Supplementary file 1 [file molecules-31-03166-s001.zip › molecules-4513180-supplementary.pdf]

Table S1. Structures and SMILES notations of designed imidazole derivatives.

Code	Structure	SMILES
**1**	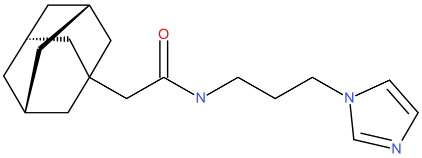	O=C(NCCCN1C=NC=C1)CC12CC3CC(CC(C3)C1)C2
**2**	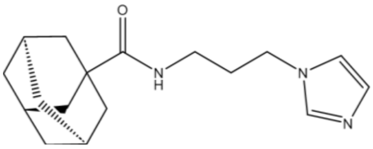	O=C(NCCCn1cncc1)C34(CC2CC(CC(C2)C3)C4)
**3**	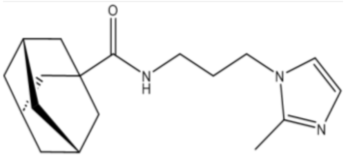	O=C(NCCCn1ccnc1C)C34(CC2CC(CC(C2)C3)C4)
**4**	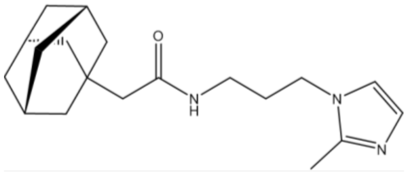	O=C(NCCCn1ccnc1C)CC34(CC2CC(CC(C2)C3)C4)
**5**	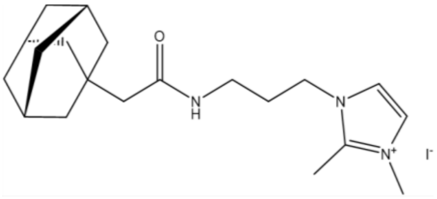	O=C(NCCCn1cc[n+](c1C)C)CC34(CC2CC(CC(C2)C<br>3)C4)

**6**	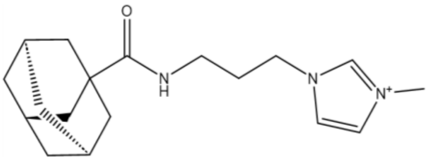	O=C(NCCCn1cc[n+](c1)C)C34(CC2CC(CC(C2)C3)C4)
**7**	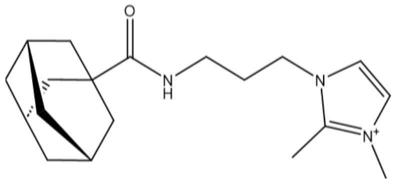	O=C(NCCCn1cc[n+](c1C)C)C34(CC2CC(CC(C2)C3)C4)
**8**	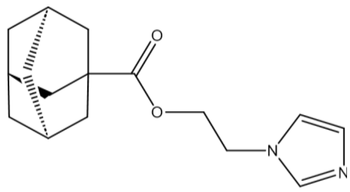	O=C(OCCn1cncc1)C34(CC2CC(CC(C2)C3)C4)
**9**	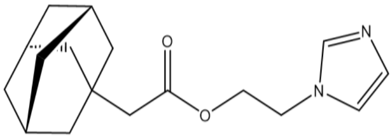	O=C(OCCn1cncc1)CC34(CC2CC(CC(C2)C3)C4)
**10**	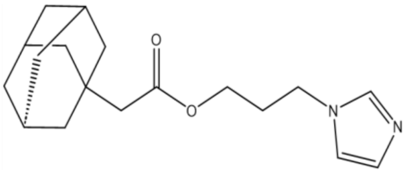	O=C(OCCCn1cncc1)CC34(CC2CC(CC(C2)C3)C4)
**11**	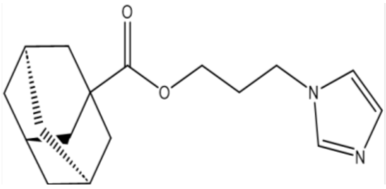	O=C(OCCCn1cncc1)C34(CC2CC(CC(C2)C3)C4)

**12**	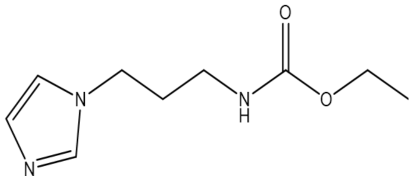	O=C(OCC)NCCCn1cncc1
**13**	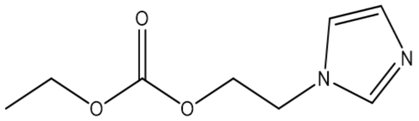	O=C(OCC)OCCn1cncc1
**14**	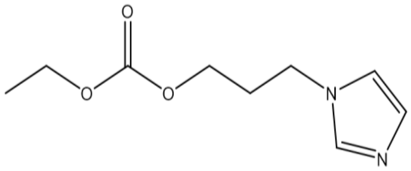	O=C(OCC)OCCCn1cncc1
**15**	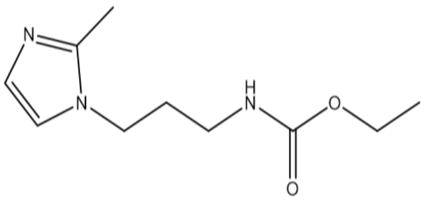	O=C(OCC)NCCCn1ccnc1C
**16**	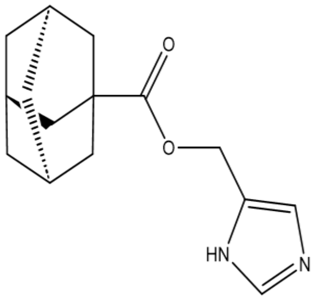	O=C(OCc1cnc[nH]1)C34(CC2CC(CC(C2)C3)C4)
**17**	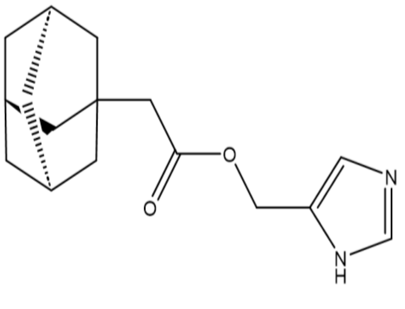	O=C(OCc1cnc[nH]1)CC34(CC2CC(CC(C2)C3)C4)

**18**	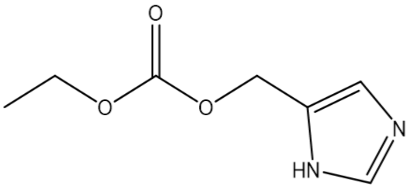	O=C(OCc1cnc[nH]1)OCC
**19**	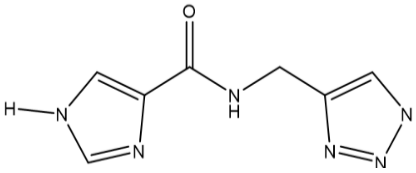	O=C(NCc1nnnc1)c2nc[nH]c2
**20**	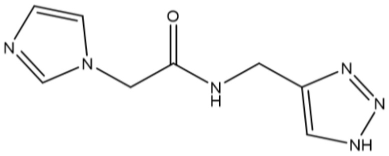	O=C(NCc1nn[nH]c1)Cn2cncc2
**21**	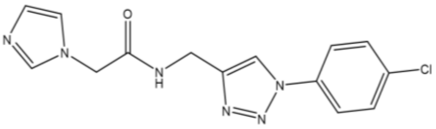	O=C(NCc1nnn(c1)c2ccc(cc2)Cl)Cn3cncc3
**22**	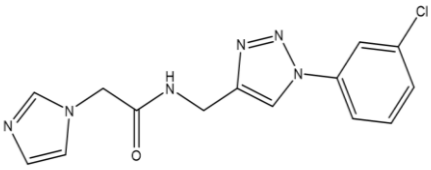	O=C(NCc1nnn(c1)c2cccc(c2)Cl)Cn3cncc3
**23**	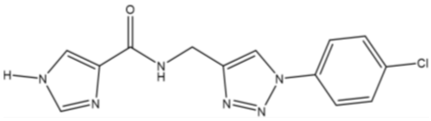	O=C(NCc1nnn(c1)c2ccc(cc2)Cl)c3nc[nH]c3
**24**	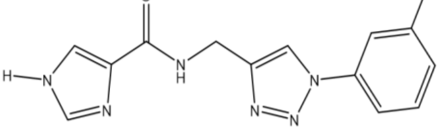	O=C(NCc1nnn(c1)c2cccc(c2)Cl)c3nc[nH]c3

**25**	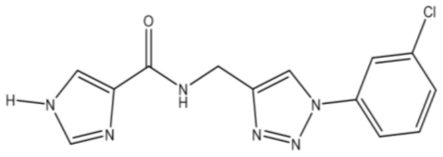	O=C(c1nc[nH]c1)n2ncnc2
**26**	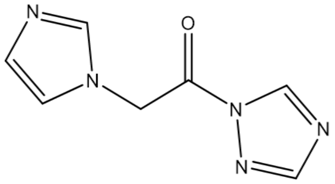	O=C(n1ncnc1)Cn2cncc2
**27**	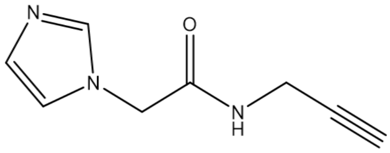	O=C(NCC#C)Cn1cncc1
**28**	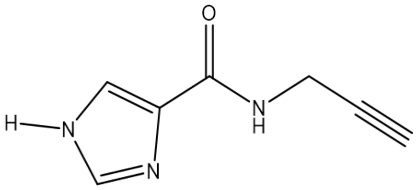	O=C(NCC#C)c1nc[nH]c1
**29**	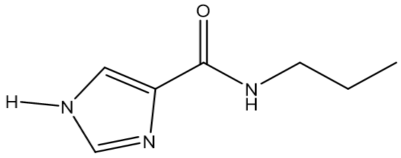	O=C(NCCC)c1nc[nH]c1
**30**	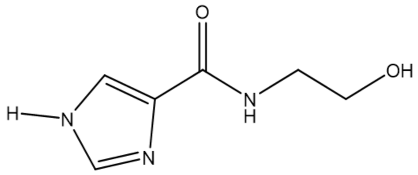	O=C(NCCO)c1nc[nH]c1
**31**	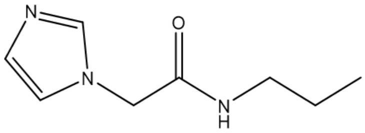	O=C(NCCC)Cn1cncc1
**32**	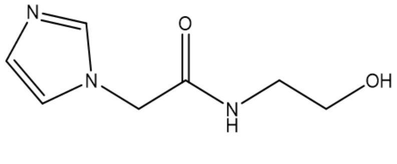	O=C(NCCO)Cn1cncc1
